# Stem cells derived exosomes as biological nano carriers for VCR sulfate for treating breast cancer stem cells

**DOI:** 10.1038/s41598-024-59736-7

**Published:** 2024-05-14

**Authors:** Ahmed H. Farouk, Ahmed Aref, Belal A. Fathy, Ahmed N. Abdallah

**Affiliations:** 1 Faculty of Biotechnology, October University for Modern Sciences and Arts, 6th of October City, Giza, Egypt; 2 Pharmacognosy Department, Pharmaceutical and Drug Industries Research Institute, National Research Centre, Dokki, Giza, Egypt; 3 Hormones Department, Medical Research and Clinical Studies Institute, National Research Centre, Dokki, Giza, Egypt

**Keywords:** Biological techniques, Biotechnology, Cancer, Stem cells, Oncology

## Abstract

Due to vincristine sulfate’s (VCR sulfate) toxicity and non-specific targeting, which might adversely damage healthy cells, its clinical application is restricted. In this study, we loaded VCR sulfate on exosomes generated from mesenchymal stem cells (MSCs) to enhance its targeted distribution. Exosomes are able to deliver molecules to specific cells and tissues and have therapeutic potential. In this study, we isolated exosomes from MSCs, and using probe-sonication approach loaded them with VCR sulfate. Using SRB assay, the cytotoxicity of VCR sulfate-Exo was assessed in T47D breast cancer cells, and the results were contrasted with those of free VCR sulfate. Then We labeled markers (CD44+/CD24−) in the cell line to assess the targeting effectiveness of VCR sulfate-Exo using flow cytometry. Our results showed that the cytotoxicity of VCR sulfate-Exo was nearly the same as that of VCR sulfate. Flow cytometry analysis revealed that VRC sulfate-Exo was more effectively targeted to MSCs than free VCR sulfate. Our study shows that loading VCR sulfate to MSCs-derived exosomes can improve their targeted delivery and lessen their side effects. Additional research is required to determine VCR sulfate-Exo’s in vivo effectiveness and safety and improve the loading and delivery strategies.

## Introduction

Cancer, one of the deadliest diseases, claims millions of lives each year and it is ranked as the second leading cause of mortality worldwide. according to the World Health Organization (WHO)^1^ Within tumors, a distinct population known as cancer stem cells (CSCs) or tumor-initiating cells (TICs) play a crucial role in tumor initiation, maintenance, metastasis, and resistance to traditional chemotherapies^2^. CSCs are stem cells that acquire mutations that enable them to bypass normal regulatory mechanisms, leading to their resistance to conventional cancer treatments like chemotherapy and radiation therapy^3^.

Stem cells, characterized by their undifferentiated and unspecialized nature, possess two key abilities: self-renewal and differentiation into mature functional cells^4^. Among the different types of stem cells, mesenchymal stem cells (MSCs) stand out for their remarkable potential to differentiate into various cell types, making them promising candidates for treating various disorders^5^.

To harness the therapeutic potential of stem cells, researchers have turned to MSC-derived exosomes (MSC-Exos), which are tiny vesicles produced by MSCs and carry a unique set of biomolecules. MSC-Exos have shown promising capabilities in enhancing tissue repair, modulating immune responses, reducing inflammation, and serving as effective drug delivery vehicles^6,7^. Compared to traditional drug delivery systems, MSC-Exos offer advantages such as small size, biocompatibility, and the ability to overcome biological barriers. Moreover, they can be engineered to target specific cells or tissues, enhancing drug selectivity and efficacy^8,9^. Numerous studies have explored the potential of MSC-Exos as drug delivery vehicles, including delivering anti-inflammatory medications for conditions like arthritis and inflammatory bowel disease, as well as therapeutic compounds for cardiovascular diseases^10^.

Vincristine sulfate (VCR sulfate) is an anticancer agent derived from Vinca Roses and is commonly used in the treatment of various cancers^11^. As part of polychemotherapy, VCR sulfate is preferred due to its limited bone marrow suppression at acceptable doses and distinct clinical effects. However, the non-specific delivery of VCR molecules can lead to side effects such as neurotoxicity^12,13^. Cancer stem cells possess ATP-binding cassette (ABC) transporters, including ABCB1, ABCC1, ABCC3, and ABCC10, which contribute to their resistance to vincristine^14^.

Breast cancer, the most commonly diagnosed cancer in women, arises from the accumulation of mutations in breast cells, leading to their malignant transformation. Rapid division and differentiation of tumor cells give rise to the formation of a lump. While breast cancer is predominantly found in women^15^, it is important to note that men can also be affected, although it accounts for only around 1% of cases^16^.

In certain cases, Vincristine sulfate (VCR sulfate) is used as a chemotherapeutic agent for breast cancer treatment^17^. However, caution must be exercised to prevent drug resistance through overdosing. The non-specific delivery of VCR molecules can lead to side effects, such as neurotoxicity^12,13^. Therefore, developing a targeted delivery system that selectively targets CSCs, particularly the resistant ones, while minimizing side effects is of paramount importance.

This study aims to investigate the targeting capacity of MSC-derived exosomes loaded with a commercially available drug, Vincristine sulfate (Hikma, UK), and compare it to the targeting capacity of the chemotherapeutic alone. The goal is to evaluate whether the exosomes can enhance the drug's targeting capabilities and reduce side effects. By specifically targeting CSCs, including the resistant subpopulation, we can improve the efficacy and safety of breast cancer treatments.

## Materials and method

### Isolation and culturing of bone marrow mesenchymal stem cells

A 3-week-old rat was euthanized using cervical dislocation. Its femur and tibia were isolated as stated in the protocol of^18^. The bone marrow was flushed from the bones into a T-25 flask containing Low glucose DMEM supplemented with 1% antibiotic and 10% FBS. The flask was incubated at 37 °C in 5% CO_2_ and 95% humidity. The culture medium was removed and replaced with fresh medium every 3–4 days. When the cells reached 70–80% confluence, they were subcultured after using 0.25% trypsin–EDTA for 3 min at 37 °C. 1ml of complete medium was used to inhibit the action of trypsin, and then recentrifuged 10 min at 200×*g*. The cell pellet was reconstituted in a complete medium and replated in a new T-25 flask^19^. This process was repeated four times, resulting in four passages of cells.

### Identification and characterization of MSCs

#### Morphological analysis

When MSC culture was on its fourth passage the morphological traits of MSCs were examined under an inverted microscope.

#### Flow cytometric analysis

After cells were washed and resuspended, the following fluorescein isothiocyanate-conjugated monoclonal antibodies were incubated in PBS supplemented with 3% FBS: anti-CD90 (554898, BD Pharmingen), 200; anti-CD73 (550,741, BD Pharmingen), and anti-CD105 (550,546, BD Pharmingen). Samples were analyzed using a forward scattering analytical method (Becton–Dickinson, Canada)^20,21^.

### MSCs derived exosomes isolation and characterization

For the extraction of exosomes, Passage IV of MSCs (5 × 106 cells/mL) was utilized. The MSCs were cultured in RPMI medium without FBS and treated with 0.5% BSA (Sigma). The cell-free supernatant was collected and centrifuged at 2000xg for 20 min to remove debris. The resulting supernatant was then subjected to further centrifugation at 100,000×*g* for 1 h at 4 °C using a Beckman Coulter Optima L 90K ultracentrifuge. This step was performed to isolate the exosomes from the remaining cellular components. Following the ultracentrifugation, the cells were washed in serum-free medium with 199 HEPES 25mM (Sigma) and underwent an additional round of ultracentrifugation using the same conditions. This process aimed to ensure the purification of the exosomes.

#### Transmission electron microscopy (TEM)

Exosomes were plated on copper grids, stained with phosphotungstic acid, and then analyzed by TEM. Secondary electronic imaging was used to capture images at a working distance of 15–25 mm and Digital acquisition and analysis were performed using a fast voltage between 20 and 30 kV using a Jeol T300 system.

#### Flow cytometry

The isothiocyanate-conjugated anti-human monoclonal antibodies used in this study included anti-CD63 (ab18235, Abcam, US) and anti-CD81 (559518, BD Pharmingen). After the extraction of exosomes, they were washed and resuspended in PBS with 3% FBS. The samples were then subjected to testing using a spray assay, specifically the Becton–Dickinson kit from Canada.

### Quantification of MSCs derived exosomes

Exosome concentration was measured using the BCA Protein Assay Kit (Novagen) according to the manufacturer’s instructions. The ratio of reagents used was 2:100, 4%Cupric Saltate: BCA solution. Exosome-enriched samples were diluted 1:10 (tenfold) in PBS, supplemented with BCA reagent, and incubated at 37 °C for 30 min before using a NanoDrop™ spectrophotometer (ND-1000, Thermo Fisher Scientific, Wilmington, 1999; 2 measurements. DE, USA) to determine the corresponding absorbance at 562 nm. A standard curve was constructed using the same procedure at bovine serum albumin concentrations ranging from 50 to 250 g/mL.

Purified Exosomes were kept overnight in the medium used for their collection then the pellet was stored at − 80℃.

### Loading of vincristine into exosomes using sonication

Vincristine (VCR sulfate) was obtained from a Drug called Vinracine (Hikma, London, United Kingdom) with a concentration of 1mg/ml. to prepare the sample 1ml of VRC was added to 1ml of Exosomes to form a total vol. 2ml of EXO- VCR loading.

Exosome-VCR loading was performed using probe sonication method^22^. EXO- VCR mixture was sonicated using probe sonication (750 v, power of 20%, 6 cycles were applied by 4-s pulse /2-s pause (average time of 24 s), left to cool on ice for 2 min, and then sonicated again. After that Incubation at 37 °C for 1h without shaking).

### Measuring loading capacity

Loading capacity was determined by using a Dialysis tubing cellulose membrane (sigma-Aldrich, Massachusetts, USA) against PBS by adding 2 ml of the mixture after probe-sonication into a cellulose dialysis sac bag that was cut off by scissors then the dialysis bag was sealed properly both from top and bottom and inserted into PBS 7.4 with 50 rpm using a shaking incubator at the room temperature. After 0.5h, the media was collected and replaced with new PBS for another 0.5h. Then the media collected and standard dilutions were filtered by syringe and tested using a Waters 2690 Alliance HPLC system equipped with a Waters 996 photodiode array detector. Standard preparation: Weight 1 mg of each standard to obtain stock solution then serial dilution to obtain conc. of (100,80,60,40,20,10 µg/ml) then filtrated using a 0.22 syringe filter and injected 10 µl. Sample preparation: 1) The sample is then filtrated using a 0.22 syringe filter and injected 10 µl. HPLC analysis conditions were: Column Kromasil C8: 4.6 × 250 mm, 5 µm, Mobile phase: 0.1% Formic acid in water: Acetonitrile, Mode of elution: isocratic, Flow rate: 1ml/min, Temperature: Ambient, Wavelength: 254 nm^22^.

### Cytotoxicity assay

T-47D: Breast ductal carcinoma cell line was obtained from the cell culture unit at Nawah Scientific Inc., (Mokatam, Cairo, Egypt). Cells were cultured in DMEM medium containing 100 mg/mL of streptomycin, 100 units/mL of penicillin, and 10% FBS in a humidified, 5% CO2 atmosphere at 37 °C.

The SRB assay was used to assess the cell viability. Aliquots of cell suspension (5 × 10–3 cells) were incubated in complete water for 24 h in 96-well plates. Another aliquot of different mediums was used to treat the cells. The cells were fixed with 150 L of 10% TCA exchanged for 1 h at 4 °C after 72 h of drug treatment. After the removal of the TCA solution, the cells were washed five times using distilled water. Aliquots of 70 L (0.4% w/v) of SRB solution were added and then incubated for 10 min in a dark atmosphere at room temperature. The plate was cleaned three times with 1% acetic acid and allowed to air dry overnight. The protein-bound SRB stain was then eluted with TRIS 150 L (10 mM), and the absorbance was determined at 540 nm using a BMG LABTECH®- FLUOstar Omega microplate reader (Ortenberg, Germany)^23,24^.

### Flowcytometric analysis of CSCs

The levels of the CD24/CD44 cells in untreated/ treated T47D breast cancer cells were evaluated using flow cytometry with a (FITC)-conjugated CD44antibody (cat no. ab27285; Abcam Inc., UK), and (APC/Cy7)-conjugated CD24 antibody (cat no. ab197137; Abcam Inc., UK). Cells were plated at a density of 6 × 10^4^ in 6-well plates for 24 h before treatment with test compounds for 48 h. Afterward, cells were mechanically harvested, centrifuged, resuspended in 500 µl PBS, and incubated with the conjugated anti-CD44-FITC and anti-CD24- APC/CY7 antibodies in a dark room at ambient temperature for 30 min. After staining, cells were injected via ACEA Novocyte™ flow cytometer (ACEA Biosciences Inc., USA). For each sample, 12,000 events were obtained, and positive FITC and/or APC/CY7 cells were measured by quadrant analysis and calculated using ACEA NovoExpress™ software (ACEA Biosciences Inc., San Diego, CA, USA).

### Statistical analysis

The findings from this study were displayed as mean ± SD and evaluated using Microsoft excel version 10 supported with Analysis Toolpak add-in at a significance level of < 0.05. One-way analysis of variance (ANOVA) was used Duncan's test was carried out to determine the similarities and differences between the control and four treated groups.

### Ethical approval

This study was approved by faculty of biotechnology ethics committee, MSA university on 18th of march, 2023. This study was reported according to ARRIVE guidelines and also Animal handling and experimentations were conducted in accordance with the Guidelines of the National Institutes of Health (NIH) regarding the care and use of animals for experimental procedures.

## Results

### Identification and characterization of MSCs

**Figure 1:**
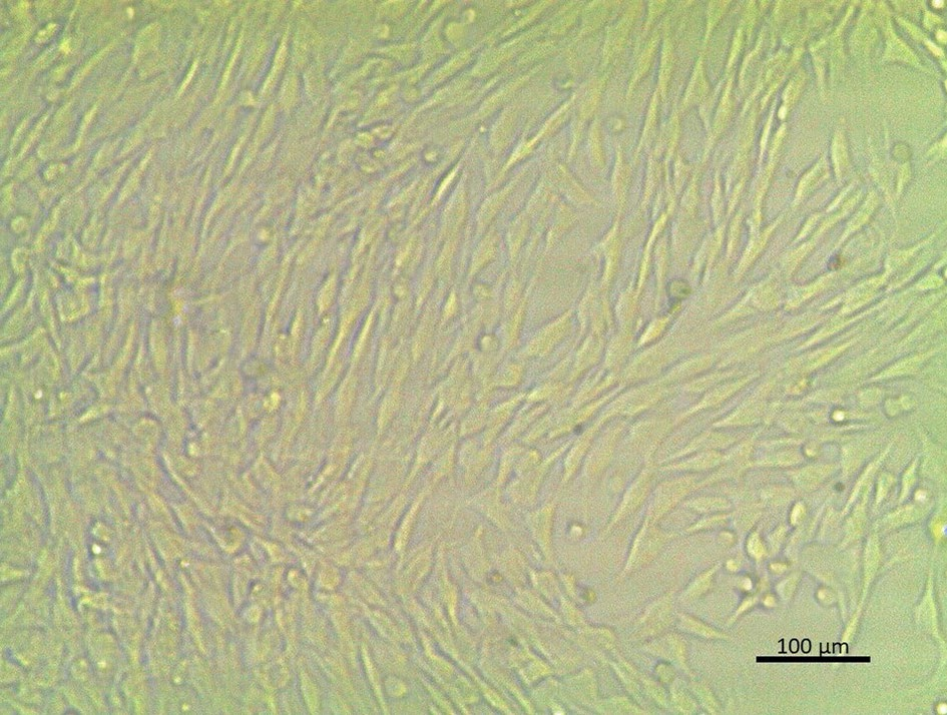
Microscopic Adaptation of Spindle-Shaped MSCs.

For identification, MSCs were observed under an inverted microscope in the fourth passage and they appeared with correct morphological traits. They were spindle-shaped and adherent as shown in (Fig. 1).

**Figure 2:**
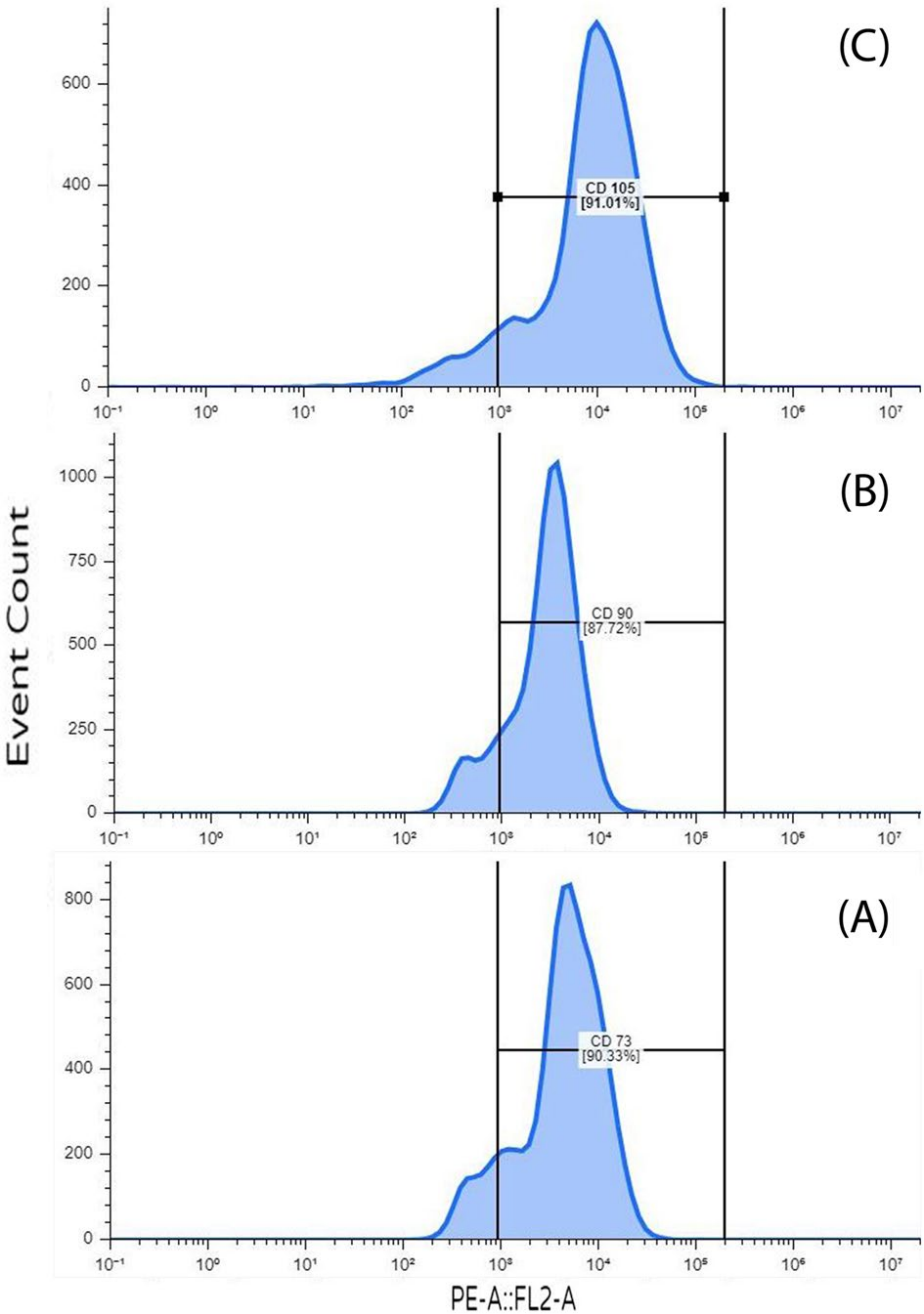
Expression levels of CD markers on MSCs. (**A**) CD73 (90.33%), (**B**) CD90 (87.72%), and (**C**) CD105 (91.01%).

Flow cytometry analysis showed positive expressions of the tested CD markers (CD73, CD90, and CD105) as it showed that CD73 is expressed in 90.33% of the sample (Fig. 2). Figure 2 also shows that around 87.72% of the marker CD90 is expressed in the sample. The marker CD105 was expressed in 91.01% of the cells in the sample as shown in Fig. 2.

### MSCs derived exosomes isolation and characterization

**Figure 3:**
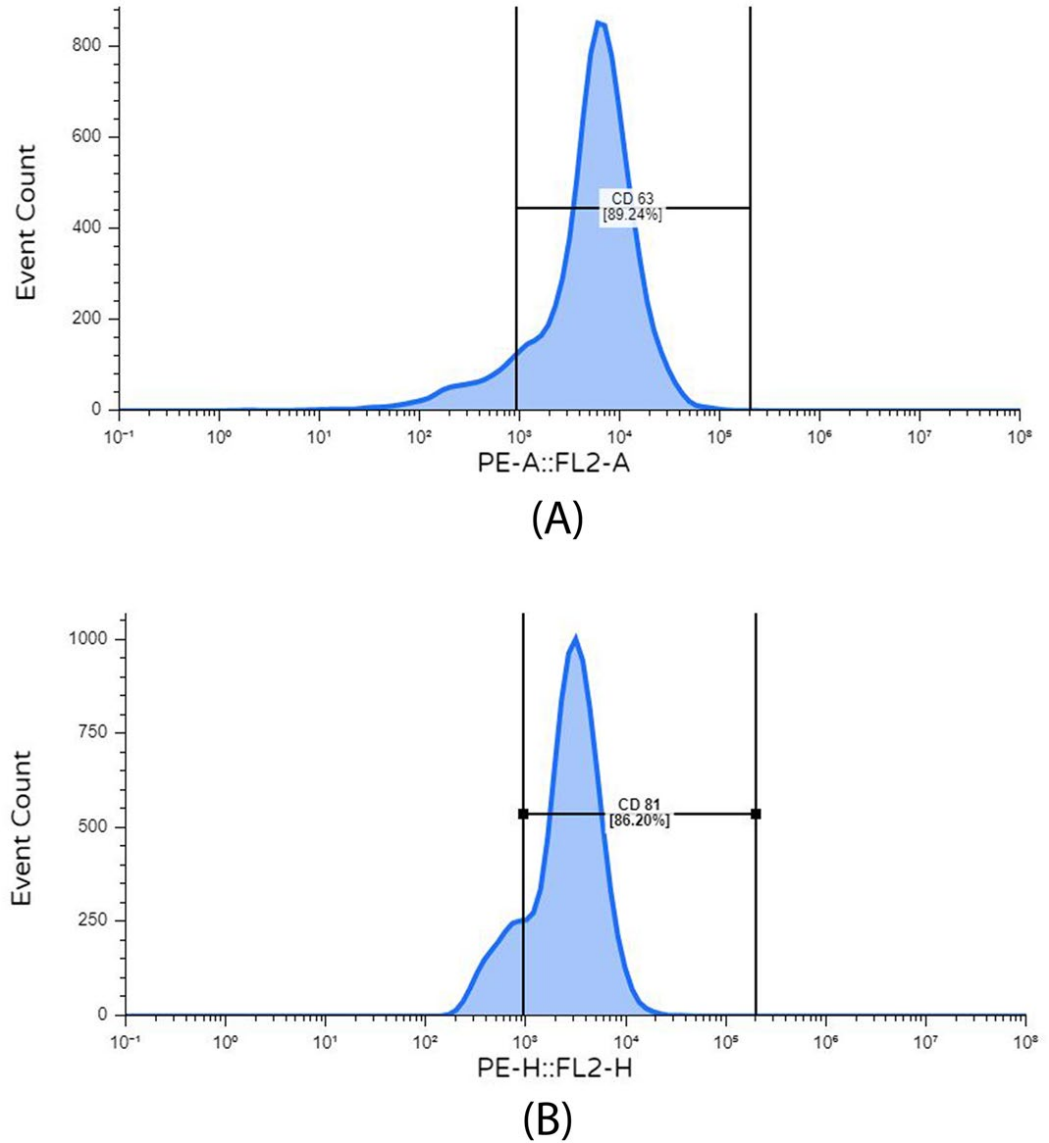
Expression levels of CD markers on MSC-derived exosomes.

MSCs derived exosomes were successfully isolated from the serum as it can be seen in their flow cytometric results and analysis that their tested markers (CD81 & CD63) showed very marked expression amounts of 86.2% for CD81 and 89.24% for CD63(Fig. 3) respectively.

**Figure 4:**
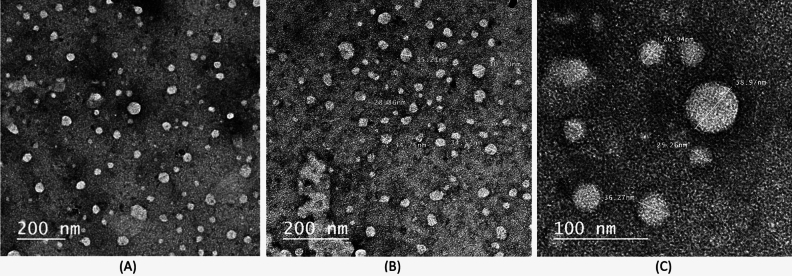
Morphological traits of exosomes under TEM under magnification power 200 nm in (**A**, **B**) and 100 nm in (**C**).

Transmission electron microscopy (TEM) results revealed the presence of microvesicles that have the characteristic and morphology of exosome, with their expected structures and with a diameter range of 25.26–58.97 nm on a 100 nm scale and 30–150 nm on a 200 nm scale as seen in (Fig. 4).

### Quantification of MSCs-derived exosomes

Measuring exosome concentration using BCA-Kit showed that the concentration of the isolated exosomes was 200 ± 20 ug/ml.

### HPLC analysis

Measuring the concentration of the unloaded drug using HPLC showed a concentration of 0.045 mg/ml. So, the amount of loaded drug = 1–0.675 mg = 0.325 mg VCR. So, the concentration of the sample is 0.1625 mg/ml. and Loading Capacity = 162.5%

### Cytotoxicity results

**Figure 5:**
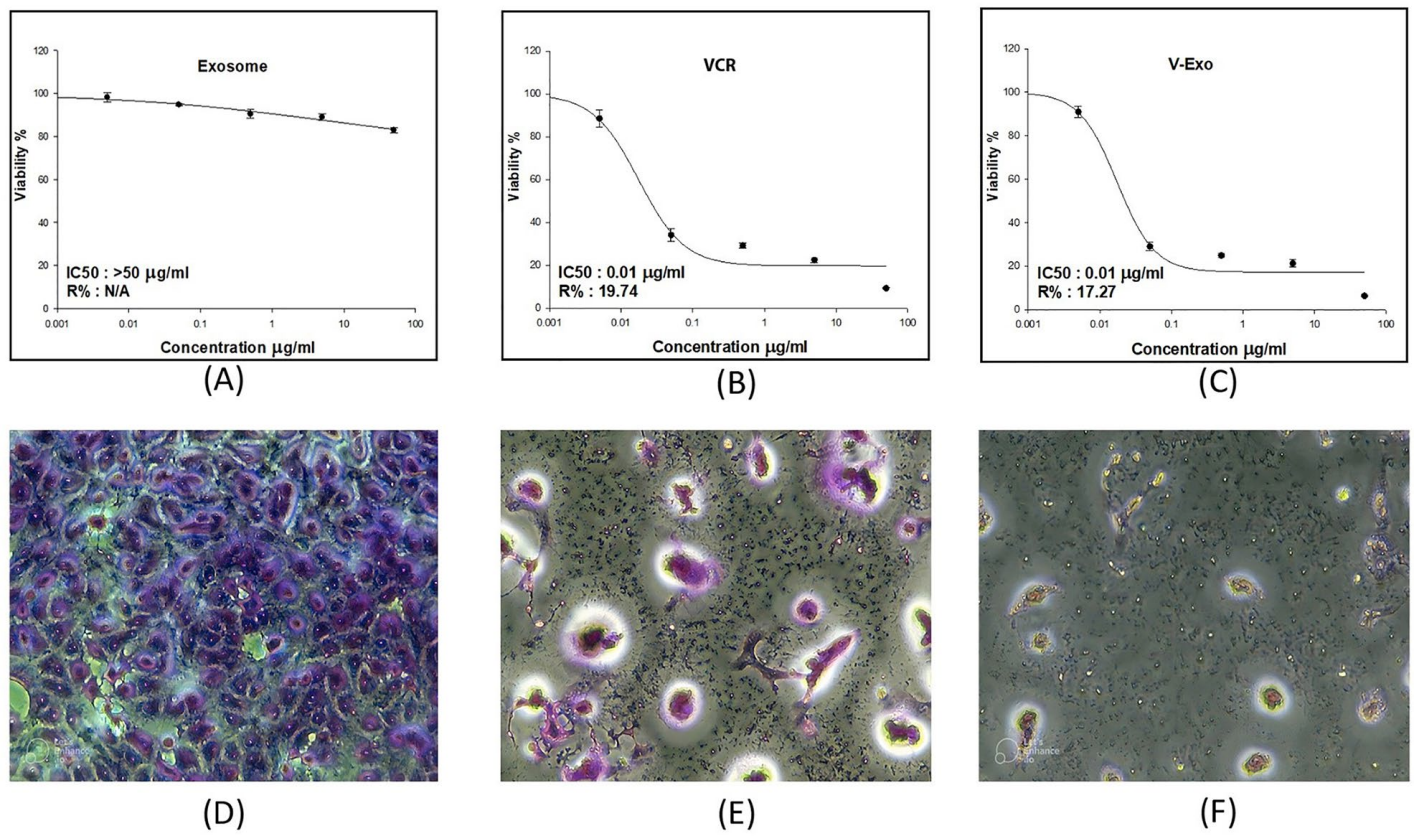
SRB Results and Morphological Traits of T47D Cells under Different Treatments for 48 Hours. (**A**) SRB results show the effects of treatment with Exosomes on T47D cells after 48 h. (**B**) Similarly, the SRB results demonstrate the impact of VCR sulfate treatment on T47D cells after 48 h. (**C**) Additionally, the SRB results reveal the outcomes of treating T47D cells with VCR sulfate-loaded exosomes for 48 h. The morphological traits of T47D cells following SRB analysis are depicted in (D) for Exosome treatment, (**E**) for VCR sulfate treatment, and (**F**) for VCR sulfate-loaded exosome treatment.

Cell viability was measured as a function of drug concentration as shown in (Fig. 5). By comparing the IC50 of VCR, EXO- VCR, and EXO the IC50 of VCR = 0.01 ug/ml IC50 of EXO- VCR = 0.01 ug/ml, and EXO > 50 ug/ml.

### Flowcytometry analysis

**Figure 6:**
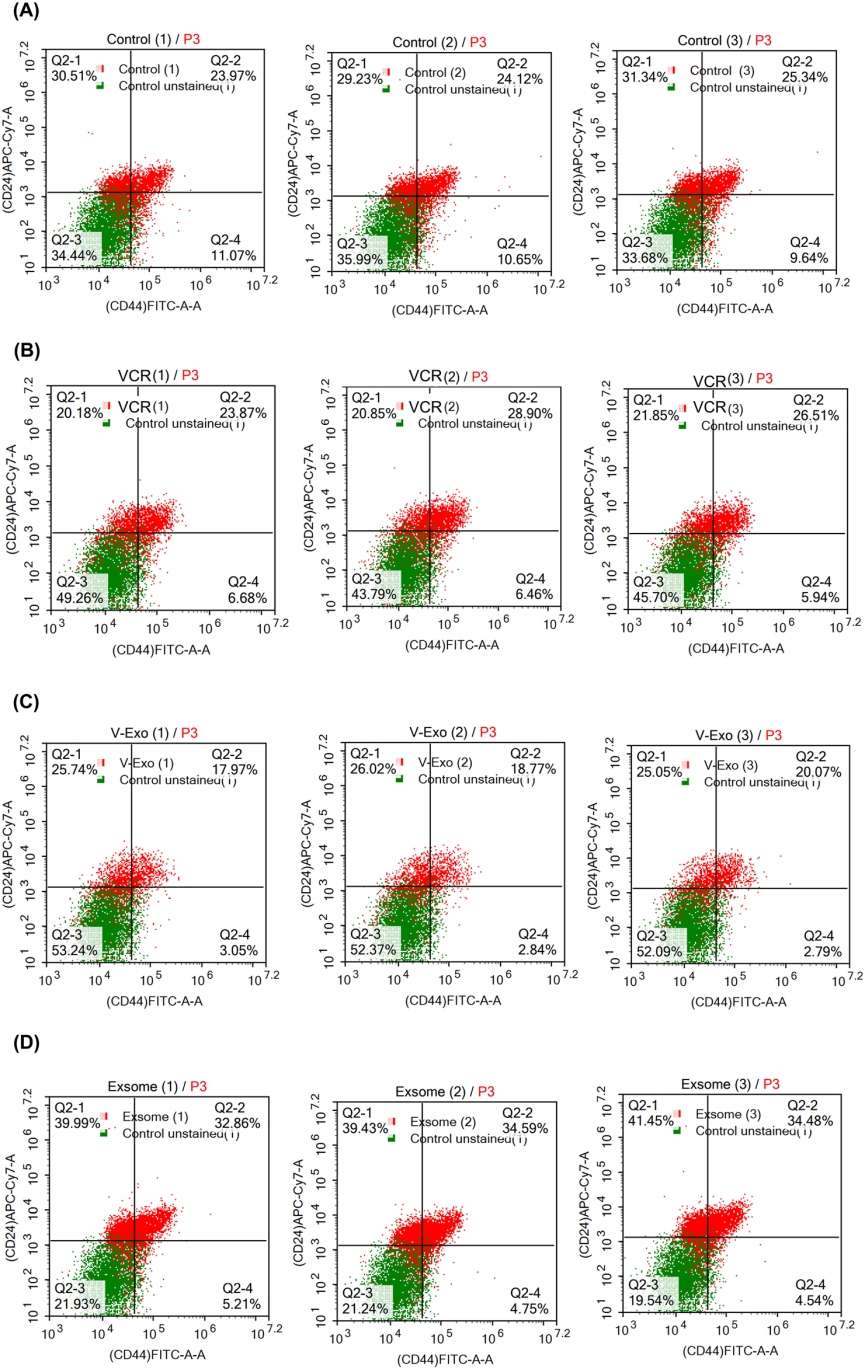
Flow Cytometric Analysis Results of T47D Cells. The flow cytometric analysis results are presented for (**A**) control T47D cells, (**B**) T47D cells treated with VCR sulfate for 48 h, (**C**) T47D cells treated with VCR sulfate-loaded exosomes for 48 h, and (**D**) T47D cells treated with exosomes for 48 h. These results provide valuable insights into the cellular characteristics and changes induced by each treatment condition, aiding in the understanding of the effects on T47D cell populations.

Figure 6 shows the results of flow cytometric analysis. In the T47D cell line, the three control samples exhibited an average expression of 10.5% of CD44 + /CD24-, which are markers commonly found in cancer stem cells (CSCs), within the total cell population. In the same figure, another group demonstrated an average expression of 6.42% of CD44 + /CD24- in the cell line. The third group displayed an average expression of 2.8% of CD44 + /CD24- in the cell line. Interestingly, the group treated with exosomes exhibited an average expression of 4.8% of CD44 + /CD24- in the cell population, as shown in both Fig. 6. Flow cytometric results show high significant difference as stated in as *P*-value > 0.05 (Table1).

**Table 1:** One way anova test that shows high Sig difference among the tested groups.

One way ANOVA						
Source of Variation	SS	df	MS	F	*P* value	F crit
Between Groups	92.7041	3	30.90136667	150.5364349	2.24316E-07	4.066180557
Within Groups	1.6422	8	0.205275			
Total	94.3463	11				

Results were analyzed using one-way analysis of variance.

## Discussion

Drug delivery systems have been widely studied in the past few years. In many different studies, Different drug delivery systems were tested to deliver different Chemotherapeutics to different types of cancers^25,26^. Exosomes may be one of the promising drug delivery systems in the future. They are extracellular vesicles that are used as signals in cell-to-cell communication in the body^27^. Lately, they have shown impressive results in delivering chemotherapeutics to specific cell types^28,29^. In this current study, the loading of Vincristine sulfate with MSC-Exos was investigated as a potential approach to enhance the targeting and specificity of the drug. Loading VCR sulfate was done using probe-sonication techniques. Due to the nano size of VCR sulfate molecules, loading was performed successfully and HPLC analysis resulted in 0.325 mg of VCR was loaded in the isolated MSC-Exos.

The developed formulation was used to treat a T47D cell line which is known to over-express receptors of CSCs (CD44 + / CD24-)^30^. After treatment 2 experiments were applied to determine the cytotoxicity of the drug and its specificity. First cytotoxicity was measured using SRB assay which showed that exosomes almost don’t have any cytotoxicity which confirms the results of^31^. The IC50 of only VCR sulfate was 0.01ug/ml which is a very high IC50 which indicates its danger for the healthy cells which made it important to enhance their delivery only for selected cells. VCR -Exos had the same IC50 of VCR which indicates that loading didn’t affect the Molecules of VCR and exosomes didn’t stop their function as well. Finally, it can be said that we had a developed formulation with the same concentration of VCR sulfate that has the same cytotoxicity.

**Figure 7:**
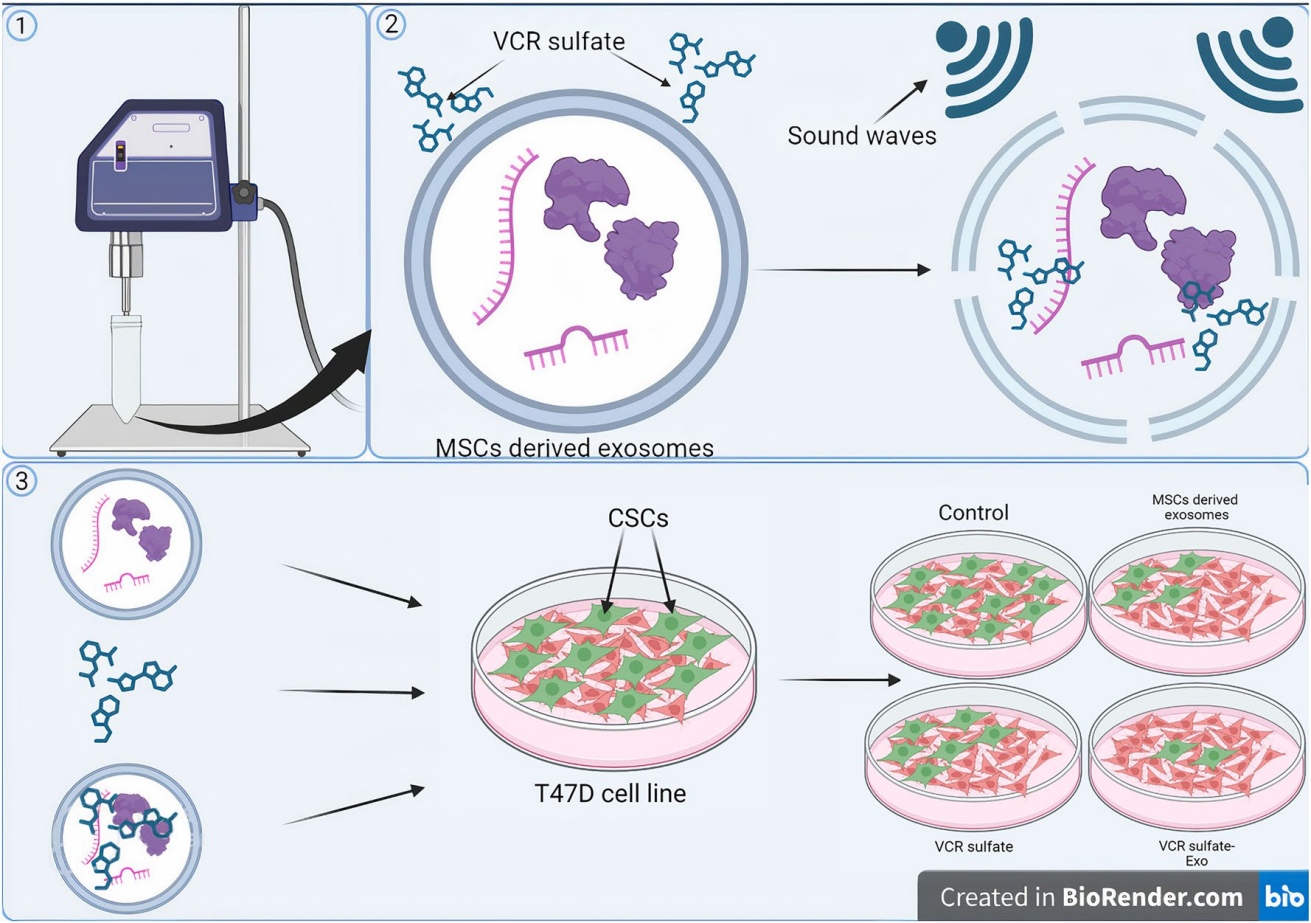
Illustration of VCR Sulfate Loading Mechanism on MSC-Derived Exosomes at the Nanoscale Using Ultrasonication Technique and the Differential Effects of MSC-Derived Exosomes, VCR Sulfate, and MSC-Derived Exosomes Loaded with VCR Sulfate on CSCs in the T47D Cell Line.

Flow cytometry was the second test applied to the T47D cell line to detect if MSC-Exos enhanced the specificity of the drug to CSCs. Flow cytometry analysis showed that the control T47D cell line Had 10.5% of CSCs which was proposed previously by^32^. MSC-Exos showed a significant decrease in the number of CSCs as it decreased the ratio of expression of CD44 + /CD24- cells from an average of 10.5% in the control to an average of 4.8% of the cells in the total amount of the cell line with almost no cytotoxicity which makes exosomes a possible cancer treatment in the future that has nearly no side effects. These results confirm the results obtained by^33^, contradictory to a few researches that stated that exosomes and MSCs derived exosomes may cause cancer progression^34,35^ so further studies dealing with exosomes and pathways they effect are needed. VCR sulfate decreased the ratio of the stem cells from an average of 10.5% to an average of 6.68% of the total expression of CD44 + /CD24-. VCR sulfate-Exos was the most effective drug among the tested 3 as it decreased the ratio of CSCs in the T47D cell line from an average of 10.5% to an average of 2.8% in the whole cell line. The previous results ensure that loading VCR sulfate on MSCs derived exosomes can enhance drug targeting and enhancement which decreases the side effects of the chemotherapeutic (Fig. 7) which confirms the results of^36,37^. That increases the chance for the exosomes to be selected as one of the very promising drug delivery systems shortly. They also may be used as a side-effect-free cancer treatment as the results showed a decrease in the number of CSCs without any symptoms of cytotoxicity.

Finally, MSC-Exos represents a promising novel method for drug delivery and regenerative medicine. MSC-Exos have already shown enormous potential in preclinical studies and hold great promise for future therapeutic uses, even though more research is necessary to fully understand and optimize their utilization, as well as to study multiple cell lines, various cancer types, and drug interactions. Further studies are also important to determine pathways that are affected by exosomes and quantification of the harmful and useful exosomes according to what they hold inside. We recommend more studies that involve in vivo experiments to recognize the actual interaction of both MSCs-derived exosomes and VCR sulfate-loaded exosomes in a realistic environment.

## Conclusion

It can be concluded that exosomes form a very good drug delivery system for harmful drugs that can affect healthy cells and can help with concentrating the drug efficiency towards the resistant cells. Our study could come out with a conclusion that MSCs derived exosomes may be useful as a drug delivery system for VCR sulfate and it can be useful as a side effects-free cancer treatment in some cases.

## Data Availability

All raw data and materials are available from the corresponding author on reasonable request. All methods that were mentioned in this manuscript were performed in accordance with the relevant guidelines and regulations.
